# 3-[(1*H*-Benzimidazol-2-yl)sulfanyl­methyl]benzonitrile

**DOI:** 10.1107/S1600536809018546

**Published:** 2009-05-23

**Authors:** Jin Rui Lin, Jia He, Yang Qian, Hong Zhao

**Affiliations:** a Ordered Matter Science Research Center, College of Chemistry and Chemical Engineering, Southeast University, Nanjing 210096, People’s Republic of China

## Abstract

In the title compound, C_15_H_11_N_3_S, the dihedral angle between the benzimidazole ring system and the benzene ring is 51.8 (2)°. The crystal structure exhibits inter­molecular N—H⋯N hydrogen bonds which lead to the formation of *C*(4) chains along the [001] direction.

## Related literature

For pharmacological activities of benzimidazole and its derivatives, see: Negwer & Scharnow (2001 ▶). For graph-set notation, see: Bernstein *et al.* (1995 ▶).
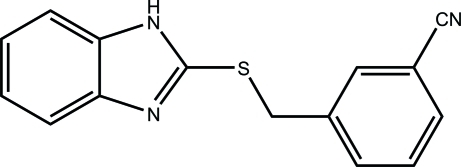


## Experimental

### 

#### Crystal data

C_15_H_11_N_3_S
*M* *_r_* = 265.33
Monoclinic, 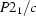

*a* = 15.384 (4) Å
*b* = 9.280 (4) Å
*c* = 9.887 (4) Å
β = 101.63 (3)°
*V* = 1382.5 (8) Å^3^
*Z* = 4
Mo *K*α radiation
μ = 0.22 mm^−1^
*T* = 292 K
0.35 × 0.30 × 0.25 mm

#### Data collection

Rigaku SCXmini diffractometer
Absorption correction: multi-scan (*CrystalClear*; Rigaku, 2005 ▶) *T*_min_ = 0.927, *T*_max_ = 0.947
12254 measured reflections
2711 independent reflections
1926 reflections with *I* > 2σ(*I*)
*R*_int_ = 0.052

#### Refinement

*R*[*F* ^2^ > 2σ(*F* ^2^)] = 0.070
*wR*(*F* ^2^) = 0.184
*S* = 1.13
2711 reflections
172 parameters
H-atom parameters constrained
Δρ_max_ = 0.23 e Å^−3^
Δρ_min_ = −0.16 e Å^−3^

### 

Data collection: *CrystalClear* (Rigaku, 2005 ▶); cell refinement: *CrystalClear*; data reduction: *CrystalClear*; program(s) used to solve structure: *SHELXS97* (Sheldrick, 2008 ▶); program(s) used to refine structure: *SHELXL97* (Sheldrick, 2008 ▶); molecular graphics: *SHELXTL* (Sheldrick, 2008 ▶); software used to prepare material for publication: *SHELXTL*.

## Supplementary Material

Crystal structure: contains datablocks I, global. DOI: 10.1107/S1600536809018546/bx2210sup1.cif

Structure factors: contains datablocks I. DOI: 10.1107/S1600536809018546/bx2210Isup2.hkl

Additional supplementary materials:  crystallographic information; 3D view; checkCIF report

## Figures and Tables

**Table 1:** Hydrogen-bond geometry (Å, °)

*D*—H⋯*A*	*D*—H	H⋯*A*	*D*⋯*A*	*D*—H⋯*A*
N2—H2*A*⋯N1^i^	0.86	1.98	2.838 (3)	174

Symmetry code: (i) 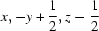.

**Fig. 1.**
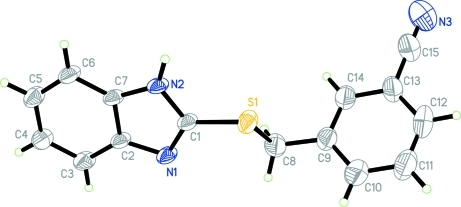
The molecular structure of the title compound, showing the atomic numbering scheme. The displacement ellipsoids are drawn at the 30% probability level.

**Fig. 2.**
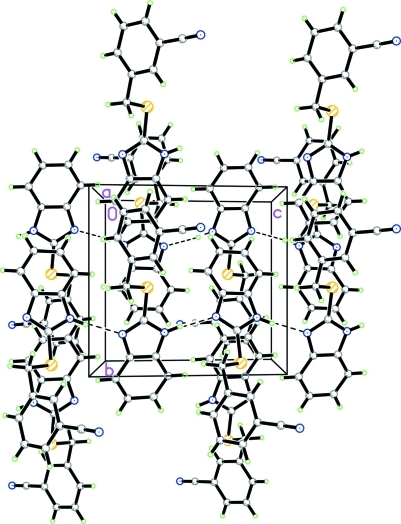
Packing diagram of the title compound, showing the structure along the a axis.

## supplementary crystallographic information

### Comment

It is well known that Benzimidazole and its derivatives have received much attention due to their versatile pharmacological activities (Negwer & Scharnow, 2001). To further investigate the influence of the bridging ligand on the formation of supramolecular complexes, we designed and prepared a Benzimidazole-containing ligand, and used the ligand to generate some coordination polymers with interesting topologies. Here, we reported the crystal structure of the title compound. The molecular structure of the title compound, C_15_H_11_N_3_S, and the atomic labeling scheme are shown in Fig. 1. In this Structure, the nine-membered benzimidazole ring system C1/N1/C2/C3/C4/C5/C6/C7/N2 is essentially planar. The phenyl ring C9/C10–C14 is connected to the benzimidazole ring system by the SCH_2_ group. The bond lengths and angles have normal values, the dihedral angle between the benzimidazole ring system and the phenyl substituent is 51.77 (22)°, and the molecules are linked by intermolecular N—H···N hydrogen bonds which leads to the formation chains C(4) along the [001] direction (Bernstein *et al.*, 1995).

### Experimental

3-(bromomethyl)benzonitrile (11 mmol) was added to 2-mercaptobenzimidazole (10 mmol) in dry ethanol (25 ml). The mixture was refluxed for 24 h. The reaction mixture was diluted with ethyl acetate (100 ml), and the resulting solid was collected and dissolved in 20 ml of water. 40 ml of a solution of sodium hydrogen carbonate (35 g in 100 ml of water) was added. A white powder was isolated by filtration and dried to give the title compound. Colourless crystals of the title compound suitble for X-ray diffraction were from a solution of 50 mg in 20 ml methanol after 7 d.

### Refinement

All H atoms were fixed geometrically and treated as riding with C—H = 0.93 Å (aromatic), 0.97 Å (methylene) or 0.96 Å (methyl) with *U*_iso_(H) = 1.2*U*_eq_(Caromatic, Cmethylene)

### Crystal data

**Table:**

C_15_H_11_N_3_S	*F*(000) = 552
*M**_r_* = 265.33	*D*_x_ = 1.275 Mg m^−^^3^
Monoclinic, *P*2_1_/*c*	Mo *K*α radiation, λ = 0.71073 Å
Hall symbol: -P 2ybc	Cell parameters from 2386 reflections
*a* = 15.384 (4) Å	θ = 2.6–27.5°
*b* = 9.280 (4) Å	µ = 0.22 mm^−^^1^
*c* = 9.887 (4) Å	*T* = 292 K
β = 101.63 (3)°	Prism, colourless
*V* = 1382.5 (8) Å^3^	0.35 × 0.30 × 0.25 mm
*Z* = 4	

### Data collection

**Table:**

Rigaku SCXmini diffractometer	2711 independent reflections
Radiation source: fine-focus sealed tube	1926 reflections with *I* > 2σ(*I*)
graphite	*R*_int_ = 0.052
Detector resolution: 13.6612 pixels mm^-1^	θ_max_ = 26.0°, θ_min_ = 2.6°
ω scans	*h* = −18→18
Absorption correction: multi-scan (*CrystalClear*; Rigaku, 2005)	*k* = −11→11
*T*_min_ = 0.927, *T*_max_ = 0.947	*l* = −12→12
12254 measured reflections	

### Refinement

**Table:**

Refinement on *F*^2^	Primary atom site location: structure-invariant direct methods
Least-squares matrix: full	Secondary atom site location: difference Fourier map
*R*[*F*^2^ > 2σ(*F*^2^)] = 0.070	Hydrogen site location: inferred from neighbouring sites
*wR*(*F*^2^) = 0.184	H-atom parameters constrained
*S* = 1.13	*w* = 1/[σ^2^(*F*_o_^2^) + (0.0802*P*)^2^ + 0.4001*P*] where *P* = (*F*_o_^2^ + 2*F*_c_^2^)/3
2711 reflections	(Δ/σ)_max_ < 0.001
172 parameters	Δρ_max_ = 0.23 e Å^−^^3^
0 restraints	Δρ_min_ = −0.16 e Å^−^^3^

### Special details

**Table:**

Geometry. All e.s.d.'s (except the e.s.d. in the dihedral angle between two l.s. planes) are estimated using the full covariance matrix. The cell e.s.d.'s are taken into account individually in the estimation of e.s.d.'s in distances, angles and torsion angles; correlations between e.s.d.'s in cell parameters are only used when they are defined by crystal symmetry. An approximate (isotropic) treatment of cell e.s.d.'s is used for estimating e.s.d.'s involving l.s. planes.
Refinement. Refinement of *F*^2^ against ALL reflections. The weighted *R*-factor *wR* and goodness of fit *S* are based on *F*^2^, conventional *R*-factors *R* are based on *F*, with *F* set to zero for negative *F*^2^. The threshold expression of *F*^2^ > σ(*F*^2^) is used only for calculating *R*-factors(gt) *etc*. and is not relevant to the choice of reflections for refinement. *R*-factors based on *F*^2^ are statistically about twice as large as those based on *F*, and *R*- factors based on ALL data will be even larger.

### Fractional atomic coordinates and isotropic or equivalent isotropic displacement parameters (Å^2^)

**Table:**

	*x*	*y*	*z*	*U*_iso_*/*U*_eq_	
C1	0.3269 (2)	0.2203 (3)	0.2414 (3)	0.0566 (8)	
C2	0.3718 (2)	0.4296 (3)	0.3180 (3)	0.0541 (8)	
C3	0.3983 (3)	0.5528 (3)	0.3966 (3)	0.0666 (9)	
H3	0.3940	0.5575	0.4890	0.080*	
C4	0.4308 (3)	0.6672 (3)	0.3330 (3)	0.0710 (10)	
H4	0.4484	0.7505	0.3834	0.085*	
C5	0.4379 (3)	0.6608 (3)	0.1944 (3)	0.0684 (9)	
H5	0.4602	0.7398	0.1546	0.082*	
C6	0.4125 (2)	0.5406 (3)	0.1158 (3)	0.0639 (9)	
H6	0.4175	0.5362	0.0237	0.077*	
C7	0.3791 (2)	0.4256 (3)	0.1795 (3)	0.0517 (7)	
C8	0.2071 (3)	0.0318 (4)	0.3261 (4)	0.0835 (11)	
H8A	0.2330	0.0408	0.4236	0.100*	
H8B	0.1646	0.1092	0.3001	0.100*	
C9	0.1622 (3)	−0.1128 (4)	0.2964 (4)	0.0772 (10)	
C10	0.1924 (3)	−0.2344 (5)	0.3728 (5)	0.0999 (14)	
H10	0.2388	−0.2259	0.4489	0.120*	
C11	0.1549 (4)	−0.3684 (5)	0.3378 (6)	0.1166 (17)	
H11	0.1762	−0.4493	0.3897	0.140*	
C12	0.0852 (4)	−0.3812 (5)	0.2247 (6)	0.1106 (16)	
H12	0.0605	−0.4711	0.1993	0.133*	
C13	0.0527 (3)	−0.2595 (4)	0.1495 (5)	0.0899 (12)	
C14	0.0911 (3)	−0.1256 (4)	0.1844 (4)	0.0814 (11)	
H14	0.0694	−0.0446	0.1332	0.098*	
C15	−0.0208 (4)	−0.2675 (6)	0.0345 (6)	0.1185 (18)	
N1	0.33875 (19)	0.2979 (3)	0.3553 (2)	0.0602 (7)	
N2	0.34898 (18)	0.2904 (2)	0.1329 (2)	0.0572 (7)	
H2A	0.3450	0.2572	0.0507	0.069*	
N3	−0.0786 (4)	−0.2759 (6)	−0.0567 (6)	0.161 (2)	
S1	0.29335 (7)	0.03901 (9)	0.22331 (9)	0.0739 (4)	

### Atomic displacement parameters (Å^2^)

**Table:**

	*U*^11^	*U*^22^	*U*^33^	*U*^12^	*U*^13^	*U*^23^
C1	0.090 (2)	0.0474 (16)	0.0342 (14)	−0.0029 (15)	0.0174 (15)	0.0013 (12)
C2	0.087 (2)	0.0441 (14)	0.0328 (14)	0.0006 (14)	0.0159 (14)	0.0015 (11)
C3	0.109 (3)	0.0535 (17)	0.0404 (16)	−0.0015 (17)	0.0226 (17)	−0.0079 (13)
C4	0.112 (3)	0.0462 (17)	0.0565 (19)	−0.0052 (18)	0.0212 (19)	−0.0082 (14)
C5	0.103 (3)	0.0458 (16)	0.0602 (19)	−0.0037 (17)	0.0247 (19)	0.0059 (15)
C6	0.101 (3)	0.0558 (18)	0.0389 (15)	−0.0017 (17)	0.0245 (16)	0.0057 (13)
C7	0.083 (2)	0.0421 (14)	0.0311 (13)	0.0052 (14)	0.0148 (13)	0.0009 (11)
C8	0.108 (3)	0.081 (3)	0.067 (2)	−0.022 (2)	0.031 (2)	−0.0129 (19)
C9	0.095 (3)	0.068 (2)	0.073 (2)	−0.016 (2)	0.027 (2)	0.0022 (19)
C10	0.104 (3)	0.094 (3)	0.100 (3)	−0.017 (3)	0.018 (3)	0.016 (3)
C11	0.119 (4)	0.081 (3)	0.146 (5)	−0.016 (3)	0.017 (4)	0.031 (3)
C12	0.123 (4)	0.065 (3)	0.149 (5)	−0.023 (3)	0.040 (4)	0.002 (3)
C13	0.101 (3)	0.074 (3)	0.095 (3)	−0.024 (2)	0.021 (3)	−0.008 (2)
C14	0.096 (3)	0.066 (2)	0.083 (3)	−0.010 (2)	0.023 (2)	−0.0007 (19)
C15	0.139 (5)	0.100 (4)	0.112 (4)	−0.041 (3)	0.018 (4)	−0.014 (3)
N1	0.104 (2)	0.0477 (13)	0.0330 (12)	−0.0047 (13)	0.0241 (13)	−0.0015 (10)
N2	0.0971 (19)	0.0497 (13)	0.0276 (11)	−0.0005 (13)	0.0189 (12)	−0.0020 (10)
N3	0.159 (5)	0.158 (5)	0.149 (5)	−0.053 (4)	−0.010 (4)	−0.015 (4)
S1	0.1199 (8)	0.0501 (5)	0.0600 (6)	−0.0158 (5)	0.0378 (5)	−0.0082 (4)

### Geometric parameters (Å, °)

**Table:**

C1—N1	1.319 (3)	C8—S1	1.829 (4)
C1—N2	1.355 (3)	C8—H8A	0.9700
C1—S1	1.758 (3)	C8—H8B	0.9700
C2—C3	1.396 (4)	C9—C10	1.385 (6)
C2—C7	1.397 (4)	C9—C14	1.396 (6)
C2—N1	1.401 (4)	C10—C11	1.386 (6)
C3—C4	1.379 (4)	C10—H10	0.9300
C3—H3	0.9300	C11—C12	1.390 (7)
C4—C5	1.398 (4)	C11—H11	0.9300
C4—H4	0.9300	C12—C13	1.389 (6)
C5—C6	1.370 (4)	C12—H12	0.9300
C5—H5	0.9300	C13—C14	1.389 (5)
C6—C7	1.390 (4)	C13—C15	1.435 (8)
C6—H6	0.9300	C14—H14	0.9300
C7—N2	1.384 (4)	C15—N3	1.135 (7)
C8—C9	1.510 (5)	N2—H2A	0.8600
			
N1—C1—N2	113.6 (3)	H8A—C8—H8B	108.7
N1—C1—S1	126.6 (2)	C10—C9—C14	119.1 (4)
N2—C1—S1	119.6 (2)	C10—C9—C8	121.8 (4)
C3—C2—C7	119.7 (3)	C14—C9—C8	119.0 (4)
C3—C2—N1	130.4 (3)	C9—C10—C11	121.2 (5)
C7—C2—N1	109.9 (2)	C9—C10—H10	119.4
C4—C3—C2	118.0 (3)	C11—C10—H10	119.4
C4—C3—H3	121.0	C10—C11—C12	119.5 (5)
C2—C3—H3	121.0	C10—C11—H11	120.2
C3—C4—C5	121.4 (3)	C12—C11—H11	120.2
C3—C4—H4	119.3	C13—C12—C11	119.8 (4)
C5—C4—H4	119.3	C13—C12—H12	120.1
C6—C5—C4	121.4 (3)	C11—C12—H12	120.1
C6—C5—H5	119.3	C12—C13—C14	120.4 (4)
C4—C5—H5	119.3	C12—C13—C15	121.6 (4)
C5—C6—C7	117.2 (3)	C14—C13—C15	118.0 (4)
C5—C6—H6	121.4	C13—C14—C9	119.9 (4)
C7—C6—H6	121.4	C13—C14—H14	120.0
N2—C7—C6	132.4 (3)	C9—C14—H14	120.0
N2—C7—C2	105.3 (2)	N3—C15—C13	179.0 (7)
C6—C7—C2	122.3 (3)	C1—N1—C2	104.3 (2)
C9—C8—S1	106.2 (3)	C1—N2—C7	107.0 (2)
C9—C8—H8A	110.5	C1—N2—H2A	126.5
S1—C8—H8A	110.5	C7—N2—H2A	126.5
C9—C8—H8B	110.5	C1—S1—C8	102.13 (15)
S1—C8—H8B	110.5		
			
C7—C2—C3—C4	0.0 (5)	C11—C12—C13—C15	−178.2 (5)
N1—C2—C3—C4	178.4 (3)	C12—C13—C14—C9	−0.6 (7)
C2—C3—C4—C5	−0.4 (6)	C15—C13—C14—C9	179.4 (4)
C3—C4—C5—C6	0.2 (6)	C10—C9—C14—C13	−1.1 (6)
C4—C5—C6—C7	0.3 (5)	C8—C9—C14—C13	175.6 (4)
C5—C6—C7—N2	−179.1 (3)	C12—C13—C15—N3	−23 (38)
C5—C6—C7—C2	−0.6 (5)	C14—C13—C15—N3	157 (37)
C3—C2—C7—N2	179.3 (3)	N2—C1—N1—C2	−0.4 (4)
N1—C2—C7—N2	0.6 (4)	S1—C1—N1—C2	175.5 (3)
C3—C2—C7—C6	0.5 (5)	C3—C2—N1—C1	−178.6 (4)
N1—C2—C7—C6	−178.2 (3)	C7—C2—N1—C1	−0.2 (4)
S1—C8—C9—C10	88.9 (4)	N1—C1—N2—C7	0.8 (4)
S1—C8—C9—C14	−87.8 (4)	S1—C1—N2—C7	−175.4 (2)
C14—C9—C10—C11	1.6 (7)	C6—C7—N2—C1	177.8 (4)
C8—C9—C10—C11	−175.0 (4)	C2—C7—N2—C1	−0.8 (3)
C9—C10—C11—C12	−0.4 (8)	N1—C1—S1—C8	41.3 (4)
C10—C11—C12—C13	−1.3 (8)	N2—C1—S1—C8	−143.1 (3)
C11—C12—C13—C14	1.9 (8)	C9—C8—S1—C1	170.0 (3)

### Hydrogen-bond geometry (Å, °)

**Table:**

*D*—H···*A*	*D*—H	H···*A*	*D*···*A*	*D*—H···*A*
N2—H2A···N1^i^	0.86	1.98	2.838 (3)	174

Symmetry codes: (i) *x*, −*y*+1/2, *z*−1/2.
